# Characterization of Strategies to Enhance the Provision of Holistic Services Across Three Community‐Based Organizations in King County, Washington, United States

**DOI:** 10.1002/jia2.70147

**Published:** 2026-10-08

**Authors:** Sarah A. Shaw, Anjuli Wagner, Alejandra Grillo Roa, Enoch, Martha Zuniga, Steven Sawyer, Richard Williams, Taylor Brown, Francis Slaughter, Taylor Rapson, David A. Katz

**Affiliations:** ^1^ Department of Global Health University of Washington Seattle Washington USA; ^2^ Independent Consultant Seattle Washington USA; ^3^ POCAAN Seattle and Federal Way Washington USA; ^4^ Entre Hermanos Seattle Washington USA; ^5^ Seattle's LGBTQ+ Center Seattle Washington USA; ^6^ Department of Health Systems and Population Health University of Washington Seattle Washington USA

**Keywords:** community‐based participatory research, community health centres, health equity, HIV, holistic health, implementation science

## Abstract

**Introduction:**

Addressing social determinants of health within HIV services is essential to achieve HIV epidemic control in the United States. Community‐based organizations (CBOs) are uniquely positioned to implement integrated services, particularly for under‐resourced and marginalized communities. However, effective strategies to support integrated services are needed for these settings. In a community‐academic partnership, we applied implementation science tools to identify, prioritize and characterize strategies supporting three CBOs’ goals to enhance integrated services and reduce health inequities in King County, Washington, United States.

**Methods:**

From November 2021 to June 2022, we facilitated nominal group technique methods with each CBO to generate strategies aligned with conditions CBOs deemed necessary for successful integration. Staff evaluated strategies’ feasibility and effectiveness using 5‐point Likert scales (Feasibility: 1 = Very hard to do with current resources to 5 = Very easy; Effectiveness: 1 = Large negative to 5 = Large positive impact). Go‐Zone plots comparing strategies’ feasibility and effectiveness scores were generated to inform implementation prioritization. We mapped strategies to Expert Recommendations for Implementing Change (ERIC) thematic clusters and Consolidated Framework for Implementation Research (CFIR) constructs.

**Results:**

This participatory process generated 118 strategies perceived as highly impactful (mean = 4.7, range = 4.6−5.0) and with mixed feasibility (mean = 3.3, range = 3.2−4.6). Strategies most frequently aimed to improve organizational structural characteristics, staff access to knowledge/information, communications, implementer and community engagement, reflecting and evaluating, and teaming. All nine ERIC thematic clusters were represented, most commonly change infrastructure, train/educate relevant groups, develop interrelationships, use evaluative and iterative strategies, and engage consumers. Highly feasible and impactful strategies often addressed knowledge access and engagement, while fewer addressed structural characteristics and teaming. We observed patterns in barrier‐strategy mapping (CFIR‐ERIC) and in strategy feasibility and impact when reviewing construct combinations.

**Conclusions:**

Implementing integrated services requires systems‐level approaches that strengthen organizational infrastructure and respond to community priorities. Despite diverse organizational contexts, a core set of determinants and strategy types emerged, suggesting their importance for integrated services. Implementation science frameworks were valuable for translating context‐specific strategies into potentially generalizable takeaways about determinants and strategy types, though they may be unnecessary for strategy and determinant identification with CBOs. Examining the feasibility and effectiveness of strategy types addressing unique combinations of determinants may strengthen barrier‐strategy mapping.

## 1 Introduction

Addressing social determinants of health (SDH) within HIV services is essential to reduce inequities among populations most impacted by HIV and reach epidemic control in the United States [1, 2, 3, 4, 5, 6, 7, 8, 9, 10]. A recent review and meta‐analysis observed better HIV care cascade and non‐HIV‐related outcomes in settings with integrated SDH services compared to settings where services were separate [11]. Additionally, integration of pre‐exposure prophylaxis (PrEP) and other services may improve PrEP uptake and adherence, reduce HIV acquisition and facilitate engagement in other health services [12, 13, 14, 15, 16]. Integrating HIV services with other programmes is often feasible and cost‐effective, highlighting health outcome and process efficiency gains at multiple health system levels [11, 17].

Integrated service delivery models are responsive to the complex and multi‐level needs of communities [4]. Community‐based organizations (CBOs) are uniquely positioned to provide such holistic and person‐centred care to under‐resourced and marginalized communities, given their historical and ongoing roles in the HIV epidemic, long‐standing relationships with communities, extensive contextual knowledge, recognition of the inherent complexity of health and wellness, and ability to provide culturally appropriate services [9, 18, 19, 20, 21]. However, strategies supporting integrated services within CBOs remain a key knowledge gap [8]. Prior work focused on traditional healthcare settings and patients, such as improving accessibility of services, increasing patient awareness regarding health topics and service availability, and training providers on SDH [5]. Research is needed to examine what systems and organizational‐level strategies facilitate integrated services within CBOs.

A community‐academic partnership between University of Washington (UW) researchers and three CBOs was supported by the US Ending the HIV Epidemic (EHE) initiative. One project applied implementation science (IS) tools to support the CBOs’ goals to enhance integrated services (integrating HIV and other social and health programming) and reduce health inequities within their diverse communities. This manuscript describes a strategy mapping analysis of CBO‐generated strategies for facilitating integrated services. We conceptualized integrated services as the innovation, as it comprises a package of interventions. We were interested in identifying specific strategies to facilitate this model. The analysis aimed to examine CBO priorities regarding integrated service delivery, identify determinants of such services and understand what strategy types can address those determinants, responding to calls to apply empirical data to further our theoretical knowledge of IS frameworks across different contexts [22]. We also reflect on using IS frameworks to characterize CBO‐generated strategies to strengthen practice‐academic partnerships by balancing theoretical rigour and real‐world implementation processes [23].

## 2 Methods

### 2.1 Overview

This community‐academic partnership focused on building relationships and consensus around enhancing integrated services, including systematically generating and prioritizing tailored structures, processes, actions and changes (referred to as strategies throughout) to facilitate these goals. We present strategy generation process results based on data collected between 15 November 2021, and 21 June 2022. A preliminary evaluation of this planning project can be found elsewhere [24]. The University of Washington Human Subjects Division (UW HSD) determined this research to be exempt from human subjects regulations. Participation was voluntary, and CBO staff received an informational consent document describing study activities and participation options per UW HSD requirements for exempt research.

### 2.2 Study Setting

This study was conducted among three CBOs in King County, Washington, United States—Entre Hermanos, Seattle's LGBTQ+ Center and POCAAN. In addition to HIV prevention and treatment services, these community‐led organizations also provide youth art programmes, restorative justice programming, housing support, non‐medical case management and legal services, among others. Their services primarily support Hispanic/Latine, Lesbian, Gay, Bisexual, Transgender, Queer/Questioning, Intersex, Asexual (LGBTQIA+), and Black, Indigenous, People of Colour (BIPOC) communities, respectively. All CBO staff were eligible to participate.

### 2.3 Nominal Group Technique

Nominal group technique (NGT) methods [25, 26] were applied to generate strategies to facilitate integrated service delivery for each CBO. Widely used in healthcare for strategy and research prioritization [27, 28], NGT is also particularly valuable for exploratory and applied research [26, 29]. This participatory, structured, consensus‐building approach applies individual and group phases to ensure all participant voices are heard and valued equally and evaluate ideas systematically [29, 30, 31]. NGT is advantageous for groups with large time demands, allowing quick generation and transparent prioritization of many diverse ideas [25, 27, 31]. Our NGT process comprised of five steps (Figure 1, adapted from [32, 33]) conducted across two in‐person meetings for CBO A and two Zoom meetings for CBOs B and C. UW staff (SS, DAK, AW) facilitated the process with each CBO. Idea generation was grounded in a previously generated Key Driver Diagram (KDD) [34], which outlined conditions necessary for successful service integration specific to each CBO (primary drivers) and an initial set of strategies to facilitate and/or create those conditions [24].

**FIGURE 1:**
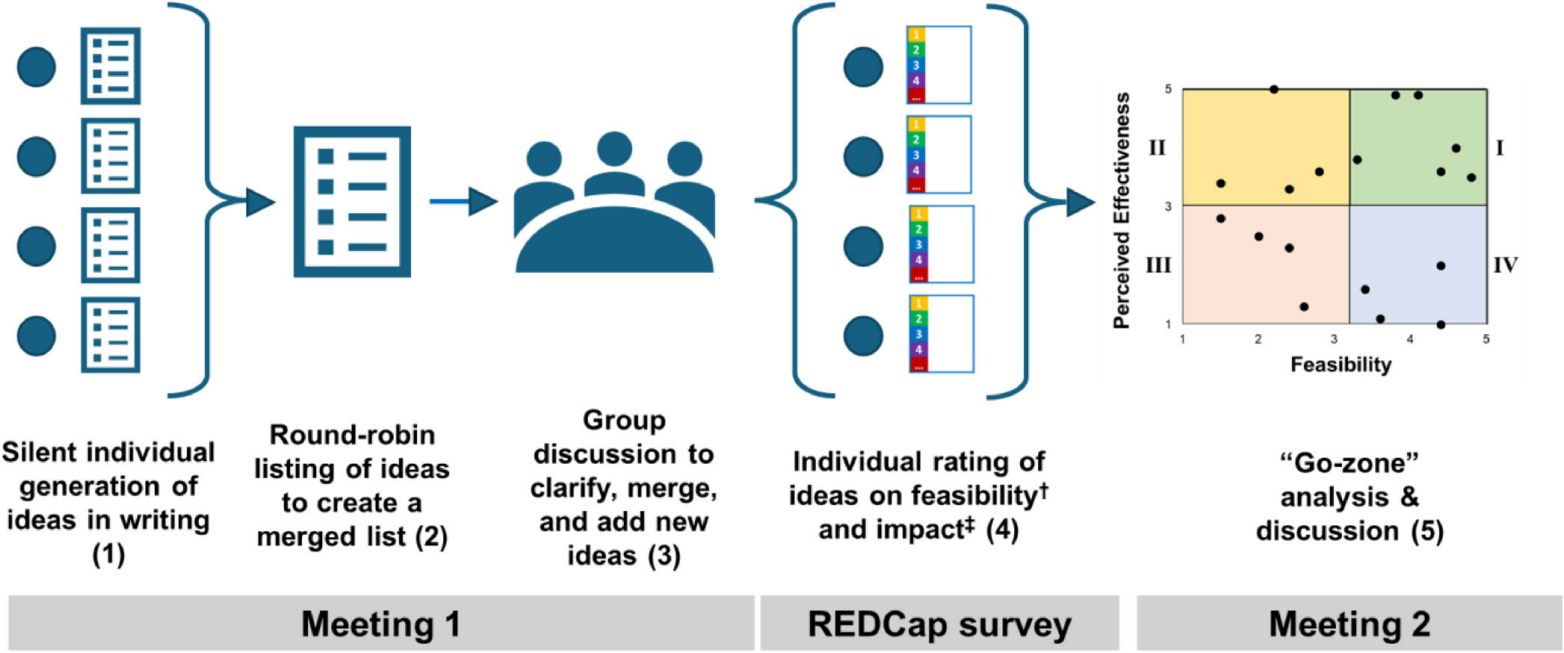
Nominal group technique procedures. **^†^** Perceived feasibility: 1 = Very hard to do with current resources, 2 = Somewhat hard, 3 = Neither hard nor easy, 4 = Somewhat easy, 5 = Very easy. **^‡^** Perceived impact: 1 = Large negative impact, 2 = Small negative impact, 3 = No impact, 4 = Small positive impact, 5 = Large positive impact.

#### 2.3.1 Strategy Evaluation

In step 4, CBO staff scored each strategy's feasibility and impact through a REDCap (Research Electronic Data Capture) [35, 36] survey. Before survey distribution, UW staff refined the strategy list based on step 3 group discussions. Informed by prior work with healthcare workers [37] and discussions with CBO leadership, feasibility was measured as “How easy would it be to do [strategy] given current resources,” and impact as “How big of an impact do you think this [strategy] would have on [organization's] goal to [organization‐specific goal from KDD].” Staff evaluated feasibility and impact using 5‐item Likert scales (Figure 1). We then generated Go‐Zone plots [33], scatterplots categorizing strategies into one of four quadrants based on mean feasibility and impact scores: (1) highly feasible/highly impactful (the “go‐zone,” strategies scoring higher than the mean on both feasibility and impact); (2) less feasible/highly impactful; (3) highly feasible/less impactful; and (4) less feasible/less impactful. We presented results to staff during the second meeting (step 5) to inform strategy prioritization.

### 2.4 Strategy Mapping

NGT‐generated strategies were mapped to constructs from the Consolidated Framework for Implementation Research (CFIR) version 2.0 [38] and thematic clusters from Expert Recommendations for Implementing Change (ERIC) [33, 39]. CBO‐generated strategies often contained multiple ideas and were not reflected in individual ERIC strategies. This mapping aimed to translate these context‐specific data into generalizable insights about multi‐level determinants that influence integrated services implementation in CBOs, types of strategies that may facilitate integrated services and inform recommendations for applying IS tools in community settings.

One UW team member (SS) mapped each strategy to ≥1 KDD primary driver, CFIR construct (implementation determinants) and ERIC thematic cluster (strategy types) [33]. This primary mapper and another UW researcher (DAK) met weekly to discuss decisions until reaching consensus, similar to qualitative codebook development. To enhance reliability, the primary mapper conducted multiple rounds of mapping, ensuring all decisions were applied consistently. We noted similar themes across organizations’ primary drivers and generated combined drivers presented here. For implementation determinants, we focused on the inner setting, outer setting and implementation process domains. Table 1 presents specific concepts with definitions and examples. We excluded individuals and innovation domains given little distinction between CFIR‐defined roles in our context, and we did not collect data on innovation perceptions, respectively. Strategies could map to multiple drivers, domains, constructs and clusters to more precisely capture strategy interconnectedness and complexity. We prioritized how CBOs conceptualized strategies; it was not feasible or appropriate to force strategies into discrete categories as external facilitators.

**TABLE 1:** Overview of concepts used for strategy mapping, associated definitions and examples of strategies aligned with each concept.

*Strategy mapping categories and associated goals*	*Concept*	*Definition/examples*
**CBO‐identified primary drivers** *Goal: Understand how CBOs define conditions and factors necessary for integrated services*	Community engagement	Includes engagement of both community members and other CBOs
	Data systems	Includes the development of tools to capture data to better support community members/clients and/or advocate for funding/services
	Inclusive and accessible environment for staff and community members	Examples include: reopening space for community groups to meet and host events, altering opening hours, ensuring spaces are accessible for all bodies and abilities, educational opportunities for staff and community, adjusting spaces to be more trauma‐informed, resources in different languages
	Staff learning	Includes training opportunities covering specific content (e.g. disability justice, trans competency and health, anti‐racist approaches, social determinants of health, organizational fundraising approaches) and skills (e.g. motivational interviewing) to ensure staff are prepared to do their jobs and work together
	Positive work environments: collaboration and communication	Collaboration and communication to improve service delivery/programmes and efficiency
	Positive work environments: defined roles and transparency	Having clear roles/expectations and transparency around decisions and hiring (e.g. standard operating procedures, training curricula, work schedules, compensation, process for new hires, need for new positions, funding)
	Positive work environments: safe space	Creating a positive and safe environment (e.g. team/relationship building, alignment of values, improving the physical space to facilitate work)
	Other	Includes strategies related to sustainability and growth of funding opportunities and partnerships and to processes for meeting state/county training requirements
**CFIR domains** *Goals: (1) identify implementation determinants each strategy aimed to address; (2) examine potential patterns in perceptions of strategies organized by determinant and relationships between constructs in this context*	Outer setting	Community members and organizations connected with the three CBOs, as well as city‐, county‐, state‐ and national‐level factors that influence the implementation of integrated services (the innovation)
	Inner setting	The CBO: organizational‐level factors (e.g. how the organization works, available resources) that influence the implementation of integrated services
	Implementation process	Processes and activities that help enhance the implementation of integrated services
**ERIC thematic clusters (33): strategy types** ^a^ *Goals: (1) categorize the types of strategies CBOs generated; (2) explore patterns in the perceptions of strategy types and alignment with CFIR determinants*	Use evaluative and iterative strategies	Develop and implement tools for quality monitoring; conduct local needs assessment; obtain and use patients/consumers and family feedback
	Provide interactive assistance	Provide local technical assistance; provide supervision
	Adapt and tailor to context	Tailor strategies; use data experts; promote adaptability
	Develop interrelationships with relevant groups and collaborators	Identify and prepare champions; inform local opinion leaders; conduct local consensus discussions; use advisory boards and workgroups
	Train/educate relevant groups and collaborators	Conduct ongoing training; develop and/or distribute educational materials; conduct educational meetings/outreach visits
	Support CBO staff	Relay data to staff; reminders for staff; revise professional roles
	Engage consumers	Involve community members/clients/patients; use mass media
	Utilize financial strategies	Access new funding; alter incentive/allowance structures
	Change infrastructure	Change record systems; change physical structure and equipment

Abbreviations: CBO, community‐based organization; CFIR, Consolidated Framework for Implementation Research; ERIC, Expert Recommendations for Implementing Change.

^a^ We present ERIC thematic clusters here with slight adaptations in language, replacing the word “stakeholders” with “relevant groups and collaborators” given its colonial and mercenary history [64, 65].

### 2.5 Analysis

R Studio (version 2023.12.1 Build 402) was used for descriptive analyses. We combined strategies across organizations, although in practice, CBOs generated and evaluated strategies independently.

We first calculated how many strategies mapped to each driver, domain, construct and thematic cluster to understand which determinants and strategy types were most common overall and for each CBO. Additionally, we assessed frequencies of combinations of determinants and of strategy types.

Next, for strategies mapping to each domain, construct and thematic cluster (hereafter collectively referred to as concepts), we calculated their distribution across the four feasibility‐impact quadrants. Strategies related to a specific concept were considered to have a typical feasibility and impact profile (e.g. highly feasible and less impactful) if ≥50% of those strategies fell within the relevant quadrant. We also evaluated feasibility and impact individually, applying a ≥75% threshold to indicate strategies related to a concept were typically considered highly or less feasible/impactful overall and for each CBO. Additionally, we calculated strategy distributions by determinant and strategy type within each quadrant.

Lastly, we reviewed combinations of CFIR constructs and ERIC thematic clusters observed across strategies. Preliminary results suggested patterns in feasibility and impact were more pronounced when evaluating strategies by determinant combinations. Therefore, we conducted a post hoc analysis examining whether certain strategy types were more common, feasible, and/or impactful for the most frequent inner setting and implementation process determinant combinations.

Results were shared with CBO teams during routine project and dedicated discussion meetings, informing strategy mapping, conference abstracts, presentations and the manuscript. Additional details on the process for generating insights and gathering manuscript feedback are available elsewhere [40].

## 3 Results

### 3.1 Overview

Approximately 40 staff across the three organizations participated in this planning project. We generated 118 strategies with 30, 36 and 52 identified by CBOs A, B and C, respectively. Strategies aligned with eight primary drivers of integrated services, with the most frequent including community engagement (*n* = 30), data systems (*n* = 30) and positive work environment: defined roles & transparency (*n* = 24) (Figure 2). Table S1 presents examples of strategies and their corresponding drivers. On average, CBO staff expected strategies to have a large positive impact on their goals to integrate service delivery (mean score = 4.7, standard deviation [SD] = 0.2, Range = 3.5−5.0) and be between “neither hard nor easy” and “somewhat easy” to implement given current resources (mean score = 3.4, SD = 0.5, Range = 1.8−4.6). Average feasibility and impact scores were similar across CBOs (feasibility range = 3.1−3.5; impact range = 4.6−4.7).

**FIGURE 2:**
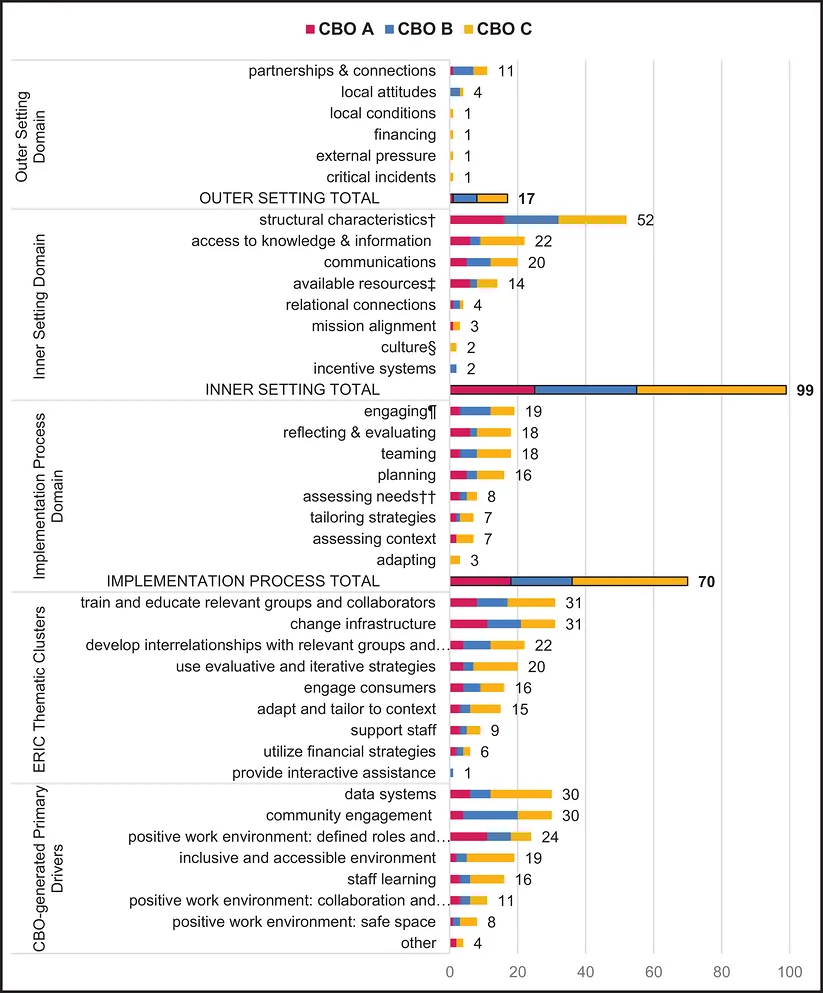
Number of strategies mapping to each construct from the Consolidated Framework for Implementation Research (CFIR) 2.0, thematic cluster from the Expert Recommendations for Implementing Change (ERIC) and primary driver generated by community‐based organization (CBO) staff. ^†^ Strategies focused on work infrastructure (48%, *n* = 25), information technology infrastructure (42%, *n* = 22) and both or broad structural characteristics (10%, *n* = 5). ^‡^ Strategies were primarily related to funding (64%, *n* = 9) and space (29%, *n* = 4). ^§^ Strategies focused on human equality‐centredness and deliverer‐centredness. ^¶^ All strategies focused on innovation recipients, one of which also involved innovation deliverers. ^††^ Majority of strategies focused on engaging innovation recipients (84%, *n* = 16).

### 3.2 Types of Strategies

This process generated strategies related to all nine ERIC thematic clusters, most commonly change infrastructure (26%, *n* = 31), train/educate relevant groups (26%, *n* = 31), develop interrelationships with relevant groups and collaborators (19%, *n* = 22), use evaluative and iterative strategies (17%, *n* = 20), and engage consumers (13%, *n* = 16) (Figure 2). These clusters were the five most common for all CBOs. The three most commonly observed combinations of strategy types were adapt and tailor to context and change infrastructure (*n* = 7), engage consumers and use evaluative and iterative strategies (*n* = 6), and engage consumers and train/educate relevant groups (*n* = 4) (Table S2).

### 3.3 Implementation Determinants

Strategies addressed determinants across all CFIR domains, with over half (56%, *n* = 66) addressing determinants in multiple domains. Strategies most frequently addressed ≥1 implementation determinant within the inner setting (84%, *n* = 99), followed by implementation process (59%, *n* = 70) and outer setting (14%, *n* = 17) domains (Figure 2). Across domains, strategies most frequently aimed to address: structural characteristics (*n* = 52), access to knowledge/information (*n* = 22), communications (*n* = 20) (all belonging to the inner setting), engaging (*n* = 19), reflecting and evaluating (*n* = 18), and teaming (*n* = 18) (implementation process). Within the outer setting, the most common determinants were partnerships and connections (65%, *n* = 11) and local attitudes (23%, *n* = 4). We observed minor variation in which constructs were most frequently addressed when comparing CBOs (Figure 2). Across 20 strategies addressing multiple inner setting determinants, we observed seven unique combinations, the most common observed for ≥2 CBOs. There were no clear patterns within the implementation process and outer setting domains (Table S2). Among 55 strategies addressing inner setting and implementation process determinants, the most common construct pairs were observed for all three CBOs (Table S3).

Table S1 provides examples of strategies addressing determinants across CFIR domains and constructs. Briefly, strategies focused on structural characteristics included improving data systems to facilitate data‐driven decision‐making across programmes and ensure clients receive individualized care. These strategies also aimed to establish positive work environments by defining clear roles and expectations for staff and facilitating transparency and autonomy regarding hiring, funding and work distribution. Strategies to increase access to knowledge/information reflected the need to invest in staff learning and included training, data access, developing standardized processes and planning, and improving collaboration across teams. Moreover, monitoring and evaluation strategies assessed both the implementation context and priorities of community members through surveys, routine community town halls and standardizing data collection elements across programmes.

### 3.4 Perceived Feasibility and Impact

There were a few patterns in feasibility or impact (separately or by combined quadrants) when organized by each concept (Table 2). Notably, strategies addressing access to knowledge/information were typically considered highly feasible (82%), whereas those addressing available resources were typically less feasible (21% highly feasible). Strategies focused on tailoring services and activities were typically highly impactful (86%). Strategies categorized as engaging consumers were often highly feasible and highly impactful (50%). No patterns emerged across CBOs (Figure S1).

**TABLE 2:** Distribution of strategies across feasibility and impact quadrants by Consolidated Framework for Implementation Research (CFIR) 2.0 constructs and Expert Recommendations for Implementing Change (ERIC) thematic clusters.

				Feasibility (F) & Impact (I) quadrant^a^					
		Feasibility	Impact	High F + High F	Low F + High I	High F + Lower I	Low F + Lower I	Highly feasible^b^	Highly impactful^b^
Concept	N (%)^c^	Mean (SD: standard deviation)	Mean (SD)	% of strategies aligned with concept				%	%
**Domain overview**									
Inner only	39 (33)	3.19 (0.58)	4.64 (0.28)	26%	23%	13%	39%	38%	49%
Inner + Outer	5 (4)	3.55 (0.38)	4.64 (0.11)	20%	20%	40%	20%	60%	40%
Inner + Process	53 (45)	3.45 (0.52)	4.68 (0.17)	26%	25%	28%	21%	55%	51%
Outer only	4 (3)	3.38 (0.15)	4.6 (0.21)	25%	0%	**50%**	25%	**▲75%** ^d^	**▼25%** ^d^
Outer + Process	6 (4)	3.63 (0.19)	4.71 (0.1)	33%	17%	0%	**50%**	33%	50%
Process only	9 (8)	3.36 (0.57)	4.64 (0.08)	44%	11%	11%	33%	56%	56%
Inner + Outer + Process	2 (2)	3.23 (0.66)	4.72 (0.21)	50%	0%	50%	0%	**▲100%** ^d^	50%
**Inner setting domain**	99 (84)			26%	23%	23%	27%		
access to knowledge & information	22 (22)	3.6 (0.39)	4.71 (0.15)	**50%**	5%	32%	14%	**▲82%**	55%
available resources	14 (14)	2.93 (0.61)	4.68 (0.23)	14%	36%	7%	43%	***▼*21%**	50%
communications	20 (20)	3.63 (0.5)	4.7 (0.18)	35%	20%	30%	15%	65%	55%
culture	2 (2)	3.01 (1.2)	4.7 (0.23)	0%	50%	50%	0%	50%	50%
incentive systems	2 (2)	3.3 (0.61)	4.7 (0.33)	0%	50%	50%	0%	50%	50%
mission alignment	3 (3)	3.27 (0.21)	4.78 (0.19)	0%	33%	33%	33%	33%	33%
relational connections	4 (4)	3.47 (0.61)	4.55 (0.27)	25%	0%	25%	**50%**	50%	***▼*25%** ^d^
structural characteristics	52 (53)	3.2 (0.53)	4.65 (0.26)	21%	27%	15%	37%	36%	48%
**Outer setting domain**	17 (14)			29%	12%	29%	29%		
critical incidents	1 (6)	3.77 (NA)	4.79 (NA)	100%	0%	0%	0%	^e^	
external pressure	1 (6)	2.62 (NA)	4.54 (NA)	0%	0%	0%	100%		
financing	1 (6)	4.29 (NA)	4.73 (NA)	100%	0%	0%	0%		
local attitudes	4 (24)	3.3 (0.14)	4.63 (0.14)	0%	**50%**	25%	25%	**▼25%** ^d^	50%
local conditions	1 (6)	3.5 (NA)	4.86 (NA)	100%	0%	0%	0%		
partnerships & connections	11 (65)	3.44 (0.31)	4.6 (0.12)	18%	9%	36%	36%	54%	27%
**Implementation process domain**	70 (59)			30%	21%	24%	24%		
adapting	3 (4)	3.04 (0.58)	4.62 (0.06)	0%	33%	33%	33%	33%	33%
assessing context	7 (10)	3.43 (0.6)	4.77 (0.17)	29%	29%	0%	43%	29%	57%
assessing needs	8 (11)	3.24 (0.33)	4.74 (0.13)	38%	25%	0%	38%	38%	63%
engaging	19 (27)	3.51 (0.41)	4.73 (0.17)	42%	21%	16%	21%	58%	63%
planning	16 (23)	3.31 (0.38)	4.73 (0.1)	38%	25%	25%	13%	63%	63%
reflecting & evaluating	18 (26)	3.3 (0.59)	4.68 (0.15)	28%	17%	17%	39%	45%	45%
tailoring strategies	7 (10)	3.37 (0.61)	4.8 (0.21)	43%	43%	0%	14%	43%	**▲86%**
teaming	18 (26)	3.59 (0.64)	4.57 (0.16)	11%	17%	39%	33%	50%	28%
**ERIC thematic clusters**									
adapt and tailor to context	15 (13)	3.33 (0.57)	4.64 (0.36)	20%	33%	13%	33%	33%	53%
change infrastructure	31 (26)	3.1 (0.59)	4.59 (0.28)	13%	23%	16%	48%	29%	36%
develop interrelationships with relevant groups and collaborators	22 (19)	3.56 (0.5)	4.64 (0.19)	23%	18%	36%	23%	59%	41%
engage consumers	16 (14)	3.47 (0.42)	4.76 (0.15)	**50%**	19%	6%	25%	56%	69%
provide interactive assistance	1 (1)	3.27 (NA)	4.67 (NA)	0%	100%	0%	0%		
support staff	9 (8)	3.36 (0.43)	4.7 (0.27)	44%	0%	22%	33%	66%	44%
train and educate relevant groups	31 (26)	3.54 (0.37)	4.71 (0.14)	48%	13%	26%	13%	74%	61%
use evaluative & iterative strategies	20 (17)	3.26 (0.62)	4.69 (0.14)	25%	25%	15%	35%	40%	50%
utilize financial strategies	6 (5)	3.04 (0.4)	4.71 (0.25)	0%	**50%**	17%	33%	**▼17%**	50%

^a^ Bolded and underlined percentages indicate that ≥50% of strategies aligned with that concept were found in the quadrant (applied only when that concept had ≥ 4 strategies).

^b^ Bolded percentages with ▲ indicate that strategies aligned with that concept were typically highly feasible or highly impactful (≥75% of strategies). Bolded percentages with ▼ indicate that strategies aligned with that concept were typically not highly feasible or impactful (≤25% strategies).

^c^ For each domain, the percentage represents the proportion of all strategies (*n* = 118) that address any construct within that domain. For each construct, the denominator is the number of strategies addressing the relevant domain.

^d^ Indicates small sample sizes (<5 strategies), suggesting the pattern should be interpreted with additional caution.

^e^ As there was only one strategy that aimed to address each of these constructs, we do not present proportions by feasibility and impact.

Examining common construct combinations and cluster combinations may reveal more consistent feasibility and impact patterns than examining concepts individually (Table 3). For example, the feasibility and impact of strategies addressing structural characteristics varied depending on the other inner setting construct with which they were aligned: strategies also addressing available resources were never highly feasible; those also addressing access to knowledge/information were typically highly feasible and highly impactful; and those addressing communications were typically highly impactful. Across inner setting and implementation process domains, strategies were typically highly feasible when focused on access to knowledge/information and planning, as well as on communications and engaging. Despite high perceived feasibility, strategies focused on communications and teaming were often considered less impactful. Alternatively, strategies addressing structural characteristics and assessing context were typically less feasible, despite high expected impact. Lastly, across strategy types, strategies categorized as adapting and tailoring to context and changing infrastructure were typically less impactful, and those related to engaging consumers and training/educating relevant groups were typically less feasible and highly impactful.

**TABLE 3:** Comparison of patterns in the feasibility and impact of strategies aligned with the most common combinations of (1) inner setting constructs^a^, (2) inner setting and implementation process constructs^a^, and (3) thematic clusters^b^ (strategy types).

	Combination	*N*	Highly feasible^c^ (%)	Highly impactful^c^ (%)
Inner setting	structural characteristics + available resources	6	*▼* **0%**	33%
	structural characteristics + access to knowledge & information	4	**▲75%**	**▲75%**
	structural characteristics + communications	4	50%	**▲75%**
Inner setting + Implementation process	access to knowledge & information + planning	9	**▲78%**	44%
	communications + engaging	8	**▲75%**	63%
	structural characteristics + teaming	8	38%	*▼* **25%**
	structural characteristics + reflecting and evaluating	7	29%	29%
	structural characteristics + planning	6	67%	**▲83%**
	communications + teaming	5	**▲80%**	*▼* **20%**
	structural characteristics + assessing context	4	*▼* **25%**	**▲75%**
ERIC thematic clusters^b^	adapt and tailor to context + change infrastructure	7	29%	*▼* **14%**
	engage consumers + use evaluative and iterative strategies	6	67%	50%
	engage consumers + train/educate relevant groups	4	*▼* **25%**	**▲75%**

^a^ Constructs are from the Consolidated Framework for Implementation Research (CFIR) 2.0.

^b^ Thematic clusters are from the Expert Recommendations for Implementing Change (ERIC).

^c^ Bolded percentages with ▲ indicate that strategies aligned with that combination of concepts were typically highly feasible or highly impactful (≥75% of strategies). Bolded percentages with ▼ indicate that strategies aligned with that combination of concepts were not typically highly feasible or impactful (≤25% strategies).

Strategies in the Go‐Zone (highly feasible, highly impactful) for all CBOs addressed access to knowledge/information and structural characteristics (inner setting), and engaging, planning and tailoring (implementation process), and were characterized as adapt and tailor to context, change infrastructure, engage consumers, train/educate relevant groups, and use evaluative and iterative strategies (Figure S2 and Table S1). Figure S3 presents the distribution of determinants by CFIR domain and of strategy types across feasibility/impact quadrants.

### 3.5 CFIR‐ERIC Matching

Each CFIR construct was nearly always observed with multiple strategy types (Figure S4), limiting the utility of reviewing 1:1 combinations of these concepts, although a few patterns emerged. For example, the majority of strategies focused on access to knowledge/information were categorized as training/educating relevant groups, and those focused on reflecting and evaluating were predominantly categorized as use evaluative and iterative strategies. Additionally, our limited data suggest certain strategy types may be more common and/or perceived as more feasible and/or impactful for specific determinant combinations (Table 4). For instance, strategies characterized as engaging consumers may be perceived as more impactful for improving communications and promoting engagement in intervention delivery compared to strategies characterized as training/educating relevant groups. This strategy type may also be more feasible for addressing structural characteristics and reflecting and evaluating, compared to strategies related to using evaluative and iterative strategies.

**TABLE 4:** Frequency and perceived feasibility and impact of strategy types organized by combined inner setting and implementation process determinant.^a^

Inner setting construct	Implementation process construct	ERIC thematic clusters	%^b^ (*n*)	High feas (%)^c^	High impact (%)^c^
access to knowledge & information	planning	train and educate relevant groups	100% (9)	**▲78%**	44%
		support staff	11% (1)		
communications	engaging	engage consumers	75% (6)	67%	**▲83%**
		train/educate relevant groups	50% (4)	**▲75%**	**▼25%**
		develop interrelationships with relevant groups	25% (2)		
		use evaluative and iterative strategies	13% (1)		
communications	assessing needs	engage consumers	100% (3)	67%	**▲100%**
		develop interrelationships with relevant groups	67% (2)		
		use evaluative and iterative strategies	33% (1)		
communications	teaming	develop interrelationships with relevant groups	80% (4)	**▲100%**	**▼25%**
		adapt/tailor to context	20% (1)		
structural characteristics	reflecting & evaluating	engage consumers	38% (3)	67%	33%
		use evaluative and iterative strategies	88% (7)	29%	43%
		adapt/tailor to context	25% (2)		
		change infrastructure	25% (2)		
structural characteristics	teaming	adapt/tailor to context	63% (5)	40%	**▼20%**
		change infrastructure	88% (7)	43%	**▼14%**
		develop interrelationships with relevant groups	13% (1)		
		provide interactive assistance	13% (1)		

Abbreviation: ERIC, Expert Recommendations for Implementing Change.

^a^ Inner setting/implementation process combinations were included in this table if they met two criteria: (1) observed in ≥ 3 strategies, and (2) more than one strategy type was aligned with the combination.

^b^ Proportion of strategies represented by the inner setting + implementation process combination that are also related to the specific ERIC thematic cluster.

^c^ Bolded percentages with ▲ indicate that strategies aligned with that combination of determinants and strategy types were typically highly feasible or highly impactful (≥75% of strategies). Bolded percentages with ▼ indicate that strategies aligned with that combination of determinants and strategy types were not typically highly feasible or impactful (≤25% strategies).

## 4 Discussion

Situated within a community‐academic partnership, this participatory process generated 118 strategies aligned with a range of CFIR determinants, representing all nine ERIC thematic clusters, and perceived as highly impactful and with mixed feasibility for facilitating integrated services to advance health equity within CBOs.

Exploring multi‐level determinants of integrated services for CBOs addresses an important gap in public health programming to reduce HIV inequities in the United States [8]. Notably, separating strategies that facilitate integrated services from those that support CBOs’ overall mission and operations was not feasible or practical. Despite unique leadership structures, programming, data collection processes and organizational cultures, a core set of determinants and strategy types emerged as particularly prominent across CBOs, suggesting their central role in organizational change to support integrated services. Go‐Zone strategies were also similar across CBOs, presenting opportunities for co‐learning and collaboration and suggesting funding priorities. These commonalities may reflect that CBOs receive the majority of HIV funding from the same state‐ and county‐based sources, have similar programmatic experience with under‐resourced communities and have a history of collaboration.

This core set of determinants aligns with those observed in other HIV service integration settings, with a few exceptions [41, 42]. Strengthening organizational infrastructure to improve data, administrative, and monitoring and evaluation processes is critical for integrated services [41, 43]. These structural changes and improved communication across implementers facilitate service alignment with community priorities and meaningful engagement of relevant parties in programme planning, foundational components of integrated services [41, 43]. Training represents an often feasible strategy [41, 43, 44], yet its effectiveness is frequently overestimated when delivered once rather than routinely [41] or without complementary strategies [45, 46]. Partnerships within the health system workforce and with external partners facilitate ongoing learning and skill development [41, 44]. Shared vision, organizational philosophy and mission alignment are key for building collaborative and motivated teams necessary for integrated services [41, 43, 44]. Unlike settings where staff were previously less involved in HIV services and often more resistant to integration [44], few strategies explicitly targeted organizational culture and mission alignment. Instead, strategies operationalized long‐embedded shared cultures around learning, supporting staff and community members, and equity within CBOs. Additionally, few strategies addressed outer setting determinants, despite the influence of economic, political and health system factors on implementation [42]. Nevertheless, feasibility and impact patterns for organizational‐level determinants may reflect broader environmental constraints. Funding restricted to specific service provision activities limits CBOs’ capacity to implement sustainable systems supporting organizational operations and staff wellbeing, and funder‐mandated data systems constrain CBOs’ access to and control over patient‐ and service‐level data.

While integrated service delivery is heavily context‐dependent [11], resulting in tailored CBO‐specific strategies, these data suggest determinants and strategy types influencing integrated services may be generalizable to similar contexts. Overall, strategies reflected the organizational systems and structures needed to implement integrated services, which may apply to integration regardless of service types. Our results substantiate the need for a system‐strengthening approach to facilitate sustainable implementation of integrated services [8], including dedicated financial investments in organizational infrastructure and human resources to improve efficiency and better support staff [8, 11, 41, 42].

Our experience using CFIR and ERIC with community partners offers critical reflections regarding theory‐driven implementation research in community‐based settings. Consistent with prior work, our results suggest IS frameworks may not always be pragmatic or necessary when working with community partners [47]. Definitions and terminology related to determinants and implementation strategies do not necessarily resonate outside academic and research settings [48], and innovation deliverers may not conceptualize them as distinct concepts [44]. While applying frameworks after strategy generation differs from best practices [49, 50], the decision to first use KDDs and NGT was grounded in community‐based participatory research principles [51, 52, 53], ensuring determinants language reflected CBOs’ cultures and staff clearly understood the strategies. Consequently, strategies aligned with context‐specific high‐priority determinants and staff could make informed decisions regarding strategies’ feasibility and impact, critical elements for optimizing strategy implementation [54, 55].

This project exemplifies an approach for merging scientific and practice priorities that centres on community perspectives. Importantly, the researchers’ deep understanding of CBO goals, processes and history strengthened the strategy mapping analysis. Ultimately, strategies addressed a comprehensive range of CFIR determinants and represented all ERIC thematic clusters without imposing IS frameworks upfront, which CBO staff felt would have been more burdensome and less accessible. Staff valued thinking critically about their challenges without being constrained by a framework, meeting partners where they were, not where researchers wanted them to be. Simultaneously, they valued the application of IS frameworks after data collection for establishing transferable lessons. IS frameworks could also be applied with community partners to bridge gaps during strategy generation by probing on additional hypothesized determinants. Mapping community‐generated strategies to IS frameworks may help prioritize strategies for testing by reviewing literature for effectiveness evidence in similar settings.

This project provides insight into barrier‐strategy matching, a prominent implementation science discussion topic [56, 57]. Evidence linking ERIC and CFIR constructs is limited [22, 58, 59, 60], and determinants frameworks have been criticized as lists of disconnected concepts [22, 59]. Existing tools attempt to facilitate barrier‐strategy mapping, including the CFIR‐ERIC matching tool [58, 61]. However, substantial heterogeneity in perceptions of ERIC strategy alignment with CFIR constructs suggests few consistent associations between strategies and determinants [58, 61]. Our results suggest barrier‐strategy mapping may be more effective when considering determinant interrelationships compared to 1:1 matching and that multiple strategies can likely address specific determinants [41]. This heterogeneity may be less satisfying to implementation scientists and clinical providers within hospital settings who often request clear implementation guidance for integrated services [4], but may better reflect CBO experiences and facilitate shared decision‐making with researchers. CBO staff expressed that generic guidance can be perceived as directive and is less helpful given their unique organizational contexts. Instead, they suggested that a menu of options to choose from would be more useful and respect their contextual expertise. Exploring whether these patterns are consistently observed in different implementation contexts will benefit future implementation science research and practice. Additionally, pairs of determinant combinations and strategy types may help elucidate mechanisms of action in different contexts, thereby supporting selection and tailoring of strategies to more effectively and efficiently influence implementation compared to generalized strategies [59, 62]. Given the rapid shift towards HIV service integration in low‐ and high‐resource settings, these results provide important community perspectives on what is necessary to ensure these efforts are sustainable and apply a rights‐based approach [63].

This exploratory analysis has limitations. While the three CBOs have diverse organizational structure and programming, our results may not be applicable to all CBOs. Perceived feasibility and impact may not reflect actual feasibility and effectiveness; however, findings informed a follow‐up study evaluating prioritized strategies in these settings. Our feasibility measure focused on implementation, given current resources and may not capture the full context. As cell counts were typically too small for statistical inference when disaggregating data by construct and CBO, we identified meaningful patterns through data visualization and summary approaches that resonated with CBOs. The thresholds we applied to identify patterns in our data may not be relevant for all analyses. In sensitivity analyses, higher thresholds identified fewer patterns but yielded similar themes. The determinants and strategy types likely do not capture the full range of perspectives from community members, clients and policy makers. Although strategy mapping relied on researcher interpretation, multiple steps were implemented to enhance rigour and ensure findings reflected CBOs’ experiences and priorities.

## 5 Conclusions

This community‐academic partnership identified implementation strategies to support integrated services within CBOs and reduce health inequities. Implementing integrated services requires systems‐level approaches that strengthen organizational infrastructure and are responsive to community priorities. This project suggests that IS frameworks may not be pragmatic when working with community partners to identify implementation determinants and strategies. Applying this theoretical lens after strategy generation proved valuable for translating context‐specific data into generalizable insights. Moving forward, IS may benefit from examining the feasibility and effectiveness of strategies addressing unique combinations of determinants within specific contexts and developing a clearer understanding of mechanisms linking determinants and strategies.

## Author Contributions

AW, MZ, SS and DAK conceptualized the study and provided supervision of study activities. SAS, AW and DAK facilitated and AGR, E, MZ, and SS participated in data collection activities. SAS, AGR, E and DAK supported project administration. SAS conducted the strategy mapping activity, led the analysis of the respective data and wrote the initial manuscript draft. All authors critically reviewed the manuscript, supported data visualization and approved the final manuscript version for submission.

## Funding

This research was funded by a 2021–22 Ending the HIV Epidemic supplement to the University of Washington/Fred Hutch Center for AIDS Research, an NIH funded programme under award number AI027757, which is supported by the following NIH Institutes and Centers: NIAID, NCI, NIMH, NIDA, NICHD, NHLBI, NIA, NIGMS, NIDDK. REDCap is supported by the Institute of Translational Health Sciences at the University of Washington, which is funded by the National Center for Advancing Translational Sciences of the National Institutes of Health under award number UL1TR002319. Additional support was provided by the University of Washington Behavioral Research Center for HIV (BIRCH), an NIMH‐funded programme (P30 MH123248).

## Conflicts of Interest

The authors declare no conflicts of interest.

## Supporting information




**Table S1**. Example strategies to support implementation of integrated services and the strategy types, implementation determinants and primary drivers with which they aligned.
**Table S2**. Frequency of unique combinations of constructs from the Consolidated Framework for Implementation Research (CFIR) 2.0 by domain and of thematic clusters from the Expert Recommendations for Implementing Change (ERIC).
**Table S3**. Frequency of unique combinations of Consolidated Framework for Implementation Research (CFIR) 2.0 inner setting and implementation process constructs across strategies and number of community‐based organizations (CBOs) for which these combinations were observed.
**Figure S1**. Comparison of patterns in perceived feasibility and impact of strategies across community‐based organizations (CBOs) organized by Consolidated Framework for Implementation Research (CFIR) 2.0 construct and Expert Recommendations for Implementing Change (ERIC) thematic cluster.
**Figure S2**. Comparison of the distribution of strategies considered highly feasible and highly impactful (Go‐Zone) by thematic cluster from the Expert Recommendations for Implementing Change (ERIC) and construct from the Consolidated Framework for Implementation Research (CFIR) 2.0 across community‐based organizations (CBOs)^†^.
**Figure S3**. Distribution of strategies aligned with domains and constructs from the Consolidated Framework for Implementation Research (CFIR) 2.0 and with thematic clusters from the Expert Recommendations for Implementing Change (ERIC) by feasibility (F) and impact (I) quadrants.
**Figure S4**. Frequency of Expert Recommendations for Implementing Change (ERIC) thematic clusters organized by Consolidated Framework for Implementation Research (CFIR) 2.0 constructs: CFIR‐ERIC matching.

## Data Availability

A subset of the data involved in this study is available in the Supplementary Materials. Additional data are available from the corresponding author upon reasonable request, following approval by the community‐based organizations.

## References

[1] W. L. Jeffries and K. D. Henny , “From Epidemiology to Action: The Case for Addressing Social Determinants of Health to End HIV in the Southern United States,” AIDS and Behavior 23, no. Suppl 3 (2019): 340–346, 10.1007/s10461-019-02687-2. PMC6858506 31563985

[2] M. Singer , N. Bulled , B. Ostrach , and E. Mendenhall , “Syndemics and the Biosocial Conception of Health,” Lancet 389, no. 10072 (2017): 941–950, 10.1016/S0140-6736(17)30003-X. 28271845

[3] P. S. Sullivan , A. Satcher Johnson , E. S. Pembleton , et al., “Epidemiology of HIV in the USA: Epidemic Burden, Inequities, Contexts, and Responses,” Lancet 397, no. 10279 (2021): 1095–1106, 10.1016/S0140-6736(21)00395-0. PMC12707558 33617774

[4] K. McKinnon , C. Lentz , D. Boccher‐Lattimore , F. Cournos , A. Pather , and S. Sukumaran , “Barriers and Facilitators to Implementing Evidence‐Based Integrated HIV and Behavioral Health Care: Perspectives From Seven Federal Ending the HIV Epidemic Jurisdictions,” AIDS Care 36, no. 11 (2024): 1555–1562. 10.1080/09540121.2024.2354897 PMC11511636 38771971

[5] P. Borkowski and N. Borkowska , “The Impact of Social Determinants of Health on Outcomes Among Individuals With HIV and Heart Failure: A Literature Review,” Cureus 16, no. 3 (2024): e55913. 10.7759/cureus.55913 PMC11003873 38601377

[6] M. De Jesus and D. R. Williams , “The Care and Prevention in the United States Demonstration Project: A Call for More Focus on the Social Determinants of HIV/AIDS,” Public Health Reports 133, no. 2_suppl (2018): 28S–33S. 10.1177/0033354918801353 PMC6262515 30457952

[7] C. Sprague and S. E. Simon , “Ending HIV in the USA: Integrating Social Determinants of Health,” Lancet 398, no. 10302 (2021): 742–743. 10.1016/S0140-6736(21)01236-8 34454663

[8] K. E. Gamarel , W. M. King , and D. Operario , “Behavioral and Social Interventions to Promote Optimal HIV Prevention and Care Continua Outcomes in the United States,” Current Opinion in HIV and AIDS 17, no. 2 (2022): 65–71. 10.1097/COH.0000000000000717 PMC8885930 35067595

[9] A. G. Robillard , C. H. Julious , S. W. Smallwood , M. Douglas , B. W. Gaddist , and T. Singleton , “Structural Inequities, HIV Community‐Based Organizations, and the End of the HIV Epidemic,” American Journal of Public Health 112, no. 3 (2022): 417–425. 10.2105/AJPH.2021.306688 PMC8887177 35196039

[10] D. Goldstein , M. Salvatore , R. Ferris , B. R. Phelps , and T. Minior , “Integrating Global HIV Services With Primary Health Care: A Key Step in Sustainable HIV Epidemic Control,” Lancet Global Health 11, no. 7 (2023): e1120–e1124. 10.1016/S2214-109X(23)00156-0 37349037

[11] C. A. Bulstra , J. A. C. Hontelez , M. Otto , A. Stepanova , E. Lamontagne , and A. Yakusik , “Integrating HIV Services and Other Health Services: A Systematic Review and Meta‐Analysis,” PLoS Medicine 18, no. 11 (2021): e1003836. 10.1371/journal.pmed.1003836 PMC8577772 34752477

[12] L. E. Mansoor , N. Yende‐Zuma , C. Baxter , K. T. Mngadi , H. Dawood , and T. N. Gengiah , “Integrated Provision of Topical Pre‐Exposure Prophylaxis in Routine Family Planning Services in South Africa: A Non‐Inferiority Randomized Controlled Trial,” Journal of the International AIDS Society 22, no. 9 (2019): e25381. 10.1002/jia2.25381 PMC6737288 31507088

[13] E. Casey , E. Kaplan‐Lewis , K. Gala , and R. Lakew , “Successful Integration of HIV PrEP in Primary Care and Women's Health Clinical Practice: A Model for Implementation,” Viruses 15, no. 6 (2023): 1365. 10.3390/v15061365 PMC10301619 37376664

[14] W. C. Sewell , V. E. Powell , M. Ball‐Burack , K. H. Mayer , A. Ochoa , and J. L. Marcus , “Brief Report: “I Didn't Really Have a Primary Care Provider Until I Got PrEP”: Patients' Perspectives on HIV Preexposure Prophylaxis as a Gateway to Health Care,” Journal of Acquired Immune Deficiency Syndromes 88, no. 1 (2021): 31–35. 10.1097/QAI.0000000000002719 PMC8369038 34397743

[15] A. Y. Liu , S. E. Cohen , E. Vittinghoff , P. L. Anderson , S. Doblecki‐Lewis , and O. Bacon , “Preexposure Prophylaxis for HIV Infection Integrated With Municipal‐ and Community‐Based Sexual Health Services,” JAMA Internal Medicine 176, no. 1 (2016): 75–84. 10.1001/jamainternmed.2015.4683 PMC5042323 26571482

[16] J. M. Sevelius , D. V. Glidden , M. Deutsch , L. Welborn , A. Contreras , and A. Salinas , “Uptake, Retention, and Adherence to Pre‐Exposure Prophylaxis (PrEP) in TRIUMPH: A Peer‐Led PrEP Demonstration Project for Transgender Communities in Oakland and Sacramento, California,” Journal of Acquired Immune Deficiency Syndromes 88, no. S1 (2021): S27–S38. 10.1097/QAI.0000000000002808 PMC8579996 34757990

[17] S. Sweeney , C. D. Obure , C. B. Maier , R. Greener , K. Dehne , and A. Vassall , “Costs and Efficiency of Integrating HIV/AIDS Services With Other Health Services: A Systematic Review of Evidence and Experience,” Sexually Transmitted Infections 88, no. 2 (2012): 85–99. 10.1136/sextrans-2011-050199 22158934

[18] K. H. Nguyen , J. D. Fields , A. G. Cemballi , R. Desai , A. Gopalan , and T. Cruz , “The Role of Community‐Based Organizations in Improving Chronic Care for Safety‐Net Populations,” Journal of the American Board of Family Medicine 34, no. 4 (2021): 698–708. 10.3122/jabfm.2021.04.200591 PMC9875859 34312263

[19] K. Chillag , K. Bartholow , J. Cordeiro , S. Swanson , J. Patterson , and S. Stebbins , “Factors Affecting the Delivery of HIV/AIDS Prevention Programs by Community‐Based Organizations,” AIDS Education and Prevention 14, no. 3 Suppl A (2002): 27–37. 10.1521/aeap.14.4.27.23886 12092934

[20] P. A. Burns , M. S. Williams , L. A. Mena , M. A. Bruce , M. Bender , and E. T. Burton , “Leveraging Community Engagement: The Role of Community‐Based Organizations in Reducing New HIV Infections Among Black Men Who Have Sex With Men,” Journal of Racial and Ethnic Health Disparities 7, no. 2 (2020): 193–201. 10.1007/s40615-019-00691-9 31942692

[21] E. Blas , L. Gilson , M. P. Kelly , R. Labonté , J. Lapitan , and C. Muntaner , “Addressing Social Determinants of Health Inequities: What Can the State and Civil Society Do?,” Lancet 372, no. 9650 (2008): 1684–1689. 10.1016/S0140-6736(08)61693-1 18994667

[22] R. Kislov , C. Pope , G. P. Martin , and P. M. Wilson , “Harnessing the Power of Theorising in Implementation Science,” Implementation Science 14 (2019): 103. 10.1186/s13012-019-0957-4 PMC6905028 31823787

[23] F. Rapport , J. Smith , K. Hutchinson , R. Clay‐Williams , K. Churruca , and M. Bierbaum , “Too Much Theory and Not Enough Practice? The Challenge of Implementation Science Application in Healthcare Practice,” Journal of Evaluation in Clinical Practice 28, no. 6 (2022): 991–1002. 10.1111/jep.13600 34268832

[24] D. Katz , S. Shaw , A. Grillo‐Roa , K. Horton , S. Sawyer , and M. Zuniga , “Evaluation of Equitable Engagement Methods in a Community‐Academic Partnership to Enhance the Provision of Holistic Services at Three Community‐Based Organizations in King County, Washington,” in 17th Annual Conference on the Science of Dissemination and Implementation (2024).

[25] A. L. Delbecq and A. H. Van de Ven , “A Group Process Model for Problem Identification and Program Planning,” Journal of Applied Behavioral Science 7, no. 4 (1971): 466–492.

[26] A. H. Van de Ven and A. L. Delbecq , “The Nominal Group as a Research Instrument for Exploratory Health Studies,” American Journal of Public Health 62, no. 3 (1972): 337–342, 10.2105/ajph.62.3.337. PMC1530096 5011164

[27] J. Allen , J. Dyas , and M. Jones , “Building Consensus in Health Care: A Guide to Using the Nominal Group Technique,” British Journal of Community Nursing 9, no. 3 (2004): 110–114. 10.12968/bjcn.2004.9.3.12432 15028996

[28] N. Harvey and C. A. Holmes , “Nominal Group Technique: An Effective Method for Obtaining Group Consensus,” International Journal of Nursing Practice 18, no. 2 (2012): 188–194. 10.1111/j.1440-172X.2012.02017.x 22435983

[29] F. Vander Laenen , “Not Just Another Focus Group: Making the Case for the Nominal Group Technique in Criminology,” Crime Science 4, no. 1 (2015): 5, 10.1186/s40163-014-0016-z.

[30] S. S. McMillan , M. King , and M. P. Tully , “How to Use the Nominal Group and Delphi Techniques,” International Journal of Clinical Pharmacy 38, no. 3 (2016): 655–662, 10.1007/s11096-016-0257-x. PMC4909789 26846316

[31] M. Gallagher , T. Hares , J. Spencer , C. Bradshaw , and I. Webb , “The Nominal Group Technique: A Research Tool for General Practice?,” Family Practice 10, no. 1 (1993): 76–81, 10.1093/fampra/10.1.76. 8477899

[32] T. Varga‐Atkins , J. McIsaac , and I. Willis , “Focus Group Meets Nominal Group Technique: An Effective Combination for Student Evaluation?,” Innovations in Education and Teaching International 54, no. 4 (2017): 289–300.

[33] T. J. Waltz , B. J. Powell , M. M. Matthieu , L. J. Damschroder , M. J. Chinman , and J. L. Smith , “Use of Concept Mapping to Characterize Relationships Among Implementation Strategies and Assess Their Feasibility and Importance: Results From the Expert Recommendations for Implementing Change (ERIC) Study,” Implementation Science 10 (2015): 1–8. 10.1186/s13012-015-0295-0 PMC4527340 26249843

[34] Available from: Shift. Key Driver Diagram (KDD) [Internet]. (2020). https://shift‐results.com/media/suso3skg/kdd.pdf.

[35] P. A. Harris , R. Taylor , R. Thielke , J. Payne , N. Gonzalez , and J. G. Conde , “Research Electronic Data Capture (REDCap)—A Metadata‐Driven Methodology and Workflow Process for Providing Translational Research Informatics Support,” Journal of Biomedical Informatics 42, no. 2 (2009): 377–381, 10.1016/j.jbi.2008.08.010. PMC2700030 18929686

[36] P. A. Harris , R. Taylor , B. L. Minor , et al., “The REDCap Consortium: Building an International Community of Software Platform Partners,” Journal of Biomedical Informatics 95 (2019): 103208, 10.1016/j.jbi.2019.103208. PMC7254481 31078660

[37] S. Hicks , B. Odhiambo , F. Abuna , J. C. Dettinger , N. Ngumbau , and L. Gómez , “Selecting Implementation Strategies to Improve Implementation of Integrated PrEP for Pregnant and Postpartum Populations in Kenya: A Sequential Explanatory Mixed Methods Analysis,” Implementation Science Communications 4, no. 1 (2023): 93. 10.1186/s43058-023-00481-9 PMC10424426 37580795

[38] L. J. Damschroder , C. M. Reardon , M. A. O. Widerquist , and J. Lowery , “The Updated Consolidated Framework for Implementation Research Based on User Feedback,” Implementation Science 17, no. 1 (2022): 1–16. 10.1186/s13012-022-01245-0 PMC9617234 36309746

[39] B. J. Powell , T. J. Waltz , M. J. Chinman , L. J. Damschroder , J. L. Smith , and M. M. Matthieu , “A Refined Compilation of Implementation Strategies: Results From the Expert Recommendations for Implementing Change (ERIC) Project,” Implementation Science 10, no. 1 (2015): 21. 10.1186/s13012-015-0209-1 PMC4328074 25889199

[40] Available from: S. Shaw and M. Zuniga , A Collaborative Manuscript‐Writing Approach: Characterization of Strategies to Enhance the Provision of Holistic Services Across Three Community‐Based Organizations in King County, Washington [Internet]. UW/Fred Hutch CFAR: Panel on Collaborative Manuscript‐Writing Within Community‐Academic Partnerships (2026). https://depts.washington.edu/cfar/events/past‐events/other‐events/.

[41] N. Watt , L. Sigfrid , H. Legido‐Quigley , S. Hogarth , W. Maimaris , and L. Otero‐García , “Health Systems Facilitators and Barriers to the Integration of HIV and Chronic Disease Services: A Systematic Review,” Health Policy and Planning 32, no. suppl_4 (2017): iv13–iv26. 10.1093/heapol/czw149 PMC5886067 28666336

[42] K. McKinnon , C. Lentz , D. Boccher‐Lattimore , F. Cournos , A. Pather , and S. Sukumaran , “Interventions for Integrating Behavioral Health Into HIV Settings for US Adults: A Narrative Review of Systematic Reviews and Meta‐Analyses, 2010–2020,” AIDS and Behavior 28, no. 8 (2024): 2492–2499. 10.1007/s10461-024-04324-z PMC11834870 38578597

[43] V. Nicolau , D. Brandão , T. Rua , and A. Escoval , “Organisation and Integrated Healthcare Approaches for People Living With HIV, Multimorbidity, or Both: A Systematic Review,” BMC Public Health [Electronic Resource] 23, no. 1 (2023): 1579. 10.1186/s12889-023-16485-y PMC10439547 37596539

[44] E. Z. Faro , J. E. Mantell , T. Gonzalez‐Argoti , S. Hoffman , Z. Edelstein , and B. Tsoi , “Implementing PrEP Services in Diverse Health Care Settings,” Journal of Acquired Immune Deficiency Syndromes 90, no. S1 (2022): S114–S128. 10.1097/QAI.0000000000002971 PMC9204802 35703763

[45] M. M. Dolcini , M. A. Davey‐Rothwell , R. R. Singh , J. A. Catania , A. A. Gandelman , and V. Narayanan , “Use of Effective Training and Quality Assurance Strategies Is Associated With High‐Fidelity EBI Implementation in Practice Settings: A Case Analysis,” Translational Behavioral Medicine 11, no. 1 (2019): 34–45. 10.1093/tbm/ibz158 PMC7877302 31773167

[46] J. M. Edmunds , R. S. Beidas , and P. C. Kendall , “Dissemination and Implementation of Evidence‐Based Practices: Training and Consultation as Implementation Strategies,” Clinical Psychology: Science and Practice 20, no. 2 (2013): 152–165. 10.1111/cpsp.12031 PMC3780425 24072959

[47] S. Dorsey , C. Johnson , C. Soi , R. D. Meza , K. Whetten , and A. Mbwayo , “Implementation Science in Plain Language: The Use of Nonjargon Terms to Facilitate Collaboration,” Implementation Research and Practice 4 (2023): 1–4, 10.1177/26334895231177474. PMC10240853 37790174

[48] K. Hoskins , A. L. Sanchez , C. Hoffacker , F. Momplaisir , R. Gross , and K. A. Brady , “Implementation Mapping to Plan for a Hybrid Trial Testing the Effectiveness and Implementation of a Behavioral Intervention for HIV Medication Adherence and Care Retention,” Frontiers in Public Health 10 (2022): 872746. 10.3389/fpubh.2022.872746 PMC9379308 35983357

[49] J. C. Moullin , K. S. Dickson , N. A. Stadnick , B. Albers , P. Nilsen , and S. Broder‐Fingert , “Ten Recommendations for Using Implementation Frameworks in Research and Practice,” Implementation Science Communications 1, no. 1 (2020): 42. 10.1186/s43058-020-00023-7 PMC7427911 32885199

[50] B. J. Powell , R. S. Beidas , C. C. Lewis , G. A. Aarons , J. C. McMillen , and E. K. Proctor , “Methods to Improve the Selection and Tailoring of Implementation Strategies,” Journal of Behavioral Health Services & Research 44, no. 2 (2017): 177–194. 10.1007/s11414-015-9475-6 PMC4761530 26289563

[51] S. E. Collins , S. L. Clifasefi , J. Stanton , K. J. E. Straits , and E. Gil‐Kashiwabara , “Community‐Based Participatory Research (CBPR): Towards Equitable Involvement of Community in Psychology Research,” American Psychologist 73, no. 7 (2018): 884–898. 10.1037/amp0000167 PMC6054913 29355352

[52] B. A. Israel , E. Eng , A. J. Schulz , and D. Satcher , Methods in Community‐Based Participatory Research for Health, 2nd ed. (John Wiley & Sons, 2013).

[53] M. Minkler and N. Wallerstein , Community‐Based Participatory Research: Implications for Public Health Funding (2003). 10.2105/ajph.93.8.1210 PMC1447939 12893597

[54] C. C. Lewis , P. A. Hannon , P. Klasnja , L. M. Baldwin , R. Hawkes , and J. Blackmer , “Optimizing Implementation in Cancer Control (OPTICC): Protocol for an Implementation Science Center,” Implementation Science Communications 2, no. 1 (2021): 44. 10.1186/s43058-021-00117-w PMC8062945 33892822

[55] R. Baker , J. Camosso‐Stefinovic , C. Gillies , E. J. Shaw , F. Cheater , and S. Flottorp , “Tailored Interventions to Address Determinants of Practice,” Cochrane Database of Systematic Reviews 2015, no. 4 (2015): CD005470. 10.1002/14651858.CD005470.pub3 PMC7271646 25923419

[56] M. Meng , Z. Wang , D. Yang , et al., “Application Status of the CFIR‐ERIC Matching Tool in Healthcare Context: A Scoping Review,” Implementation Science 21, no. 1 (2026): 23, 10.1186/s13012-026-01490-7. PMC13011528 41699613

[57] L. E. Balis and B. Houghtaling , “Matching Barriers and Facilitators to Implementation Strategies: Recommendations for Community Settings,” Implementation Science Communications 4 (2023): 144, 10.1186/s43058-023-00532-1. PMC10664260 37990243

[58] T. J. Waltz , B. J. Powell , M. E. Fernández , B. Abadie , and L. J. Damschroder , “Choosing Implementation Strategies to Address Contextual Barriers: Diversity in Recommendations and Future Directions,” Implementation Science 14, no. 1 (2019): 42. 10.1186/s13012-019-0892-4 PMC6489173 31036028

[59] R. D. Meza , J. C. Moreland , M. D. Pullmann , P. Klasnja , C. C. Lewis , and B. J. Weiner , “Theorizing Is for Everybody: Advancing the Process of Theorizing in Implementation Science,” Frontiers in Health Services 3 (2023): 1134931. 10.3389/frhs.2023.1134931 PMC10012624 36926499

[60] B. Weiner , Prioritizing Implementation Barriers: A Toolkit for Designing and Implementation Initiative (2023).

[61] Available from: CFIR Research Team‐Center for Clinical Management Research , Strategy Design—CFIR‐ERIC Implementation Strategy Matching Tool [Internet] (2023). https://cfirguide.org/choosing‐strategies/.

[62] C. C. Lewis , P. Klasnja , A. R. Lyon , B. J. Powell , R. Lengnick‐Hall , and G. Buchanan , “The Mechanics of Implementation Strategies and Measures: Advancing the Study of Implementation Mechanisms,” Implementation Science Communications 3, no. 1 (2022): 114. 10.1186/s43058-022-00358-3 PMC9588220 36273224

[63] J. Ratevosian , L. Bekker , R. Eakle , et al., “Integrating HIV Services in an Era of Global Change,” Journal of the International AIDS Society 29, no. 3 (2026): e70087, 10.1002/jia2.70087. PMC12971604 41804038

[64] M. Reed , Fast Track Impact. Should We Banish the Word “Stakeholder.” (2022).

[65] J. M. Sharfstein , “Banishing “Stakeholders”,” Milbank Quarterly 94, no. 3 (2016): 476–479. 10.1111/1468-0009.12208 PMC5020149 27620681

