# Supplementary material for: Characterization of Strategies to Enhance the Provision of Holistic Services Across Three Community‐Based Organizations in King County, Washington, United States
Source: J Int AIDS Soc. 2026 Oct 8;29(Suppl 5):e70147. doi: 10.1002/jia2.70147 (PMC13650408; doi:10.1002/jia2.70147)
Supplement: Supplementary file 1 [file JIA2-29-e70147-s001.docx]

| APPENDICES  Appendix Table 1. Example strategies to support implementation of integrated services and the strategy types, implementation determinants, and primary drivers with which they aligned. | | | | | | |
| --- | --- | --- | --- | --- | --- | --- |
|  | **CFIR^†^ constructs**  (determinants) | | | |  |  |
| ERIC^‡^ thematic cluster(s)  (Strategy type(s)) | **Inner setting** | **Implementation process** | | **Outer setting** | **CBO^§^-generated primary driver(s)** | **Strategy / Go-Zone^¶^** |
| Use evaluative and iterative strategies | - | Assessing context  Assessing needs: innovation recipients | | - | Community engagement | Conduct a community mapping activity to understand which communities we are not reaching and what support would be needed to create access to our programs and space |
|  | - | Reflecting & evaluating | | Financing | Data systems | Use data to advocate for specific programming and/or funding ► |
|  | Available resources: funding |  |  | - | Other | Evaluate the health of existing and future fundraising/funding partnerships |
| Adapt/tailor to context | - | Tailoring strategies | | - | Inclusive and accessible environment for staff and community members | Increase number of resources for Spanish-speaking clients ► |
|  | Communications  Mission alignment | - | - | |  | Ensure marketing/outreach activities are reflective of all the communities we want to serve, prioritizing BIPOC; trans, gender non confirming & femme people; young people; and LGBTQ+ non-English speaking immigrant communities |
| Train and educate relevant groups | Access to knowledge & information | Teaming  Planning | | - | Positive work environments: defined roles & transparency | Develop team standards and clear expectations so everyone is on the same page ► |
|  |  | - | | - | Staff learning | Provide staff training in motivational interviewing and strategies to make sure client has understood plan for services |
|  |  | - | | - | Data systems | Train staff on data systems, tools, and software ► |
|  | Communications | Teaming | | - | Positive work environments: defined roles & transparency | Hold monthly all staff meetings to share wins, challenges, and progress, as well as discuss workload and resources across programs |
|  | Structural characteristics: work infrastructure | Planning | | - | Staff learning | Develop standardized staff training requirements for onboarding related to social determinants of health ► |
| Utilize financial strategies | Available resources: funding | - | | - | Positive work environments: collaboration and communication  Other | Identify long term and sustainable funding opportunities |
|  |  |  | |  |  |  |
| Develop interrelationships with relevant groups and collaborators | Communications  Relational connections | Assessing context  Teaming | | - | Positive work environments: collaboration and communication | Organize meetings across departments to talk about how to improve collaborations and assess program gaps ► |
|  | Structural characteristics: work infrastructure | - | | Partnerships & connections | Community engagement | Support staff to engage in city, county, state, community level conversations & decision-making around health inequities to provide insight about serving BIPOC communities |
|  | Relational connections | Teaming | | - | Positive work environments: safe space | Organize quarterly or bi-annual teambuilding activities  Organize retreats with a focus on building relationships and sharing ideas |
|  | Culture | - | | - |  | Establish shared definitions of values communicated by organization (ex: accessibility, inclusivity, meaningful, accountability, safe) |
| Change infrastructure | Communications  Structural characteristics: work infrastructure | - | | - | Positive work environments: defined roles & transparency | Establish a transparent, equitable pay structure and increase transparency around budget and human resources |
|  | Available resources: funding  Structural characteristics: work infrastructure | - | | - |  | Hire additional staff with dedicated funding (e.g. night/weekend shift, community navigator, fluent Spanish speaker, office manager, capacity-building and education team) |
|  | Available resources: space | - | | - | Inclusive and accessible environment for staff and community members | Reopen library as space for community members ► |
| Develop interrelationships with relevant groups and collaborators  Use evaluative and iterative strategies | - | Adapting  Reflecting & evaluating  Teaming | | Partnerships & connections | Data systems  Positive work environments: collaboration and communication | Track community partnerships organization-wide to support accountability, identify gaps/challenges, and improve communication |
| Develop interrelationships with relevant groups and collaborators  Train and educate relevant groups | Access to knowledge and information | - | | Partnerships & connections | Staff learning | Bring other CBOs to share their expertise with our staff (i.e.maintaining safety, gender-affirming care) |
| Develop interrelationships with relevant groups and collaborators  Engage consumers | Communications | Assessing needs: innovation recipients  Engaging: innovation recipients | | - | Community engagement | Organize community town halls to invite community members into the new space, engage communities on specific topics, and learn what our communities need from us |
| Develop interrelationships with relevant groups and collaborators  Provide interactive assistance | Structural characteristics: information technology infrastructure | Teaming | | - | Positive work environments: collaboration and communication | Create a centralized space for programs to share upcoming events and note opportunities for other programs to attend |
| Engage consumers  Use evaluative and iterative strategies | Structural characteristics: information technology infrastructure | Adapting  Reflecting & evaluating | | - | Community engagement  Data systems | Develop a system to review and adapt service delivery based on community feedback |
|  |  | Assessing context  Engaging: innovation recipients  Reflecting & evaluating | | - | Community engagement  Inclusive and accessible environment for staff and community members | Establish a feedback mechanism for community members to report if our space is not safe or accessible without creating more undue harm |
|  | Communications | Assessing needs: innovation recipients  Engaging: innovation recipients | | - | Community engagement | Hold quarterly public info sessions to understand community needs and concerns ► |
| Adapt/tailor to context  Use evaluative and iterative strategies | Structural characteristics: information technology infrastructure | Reflecting & evaluating | | - | Data systems | Establish a central comprehensive framework for data collection & review across programs |
| Train and educate relevant groups  Use evaluative and iterative strategies | Access to knowledge and information | Reflecting & evaluating | | - | Inclusive and accessible environment for staff and community members  Staff learning | Educate all staff about gender-affirming care and review all programs to ensure sure they are provided in a gender-affirming way ► |
| Support clinicians  Train and educate relevant groups | Access to knowledge & information  Structural characteristics: work infrastructure | - | | - | Positive work environments: defined roles & transparency | Map support roles for managing programs so that staff understand where to go when they have questions/needs ► |
| Change infrastructure  Engage consumers | - | Assessing needs: innovation recipients  Engaging: innovation  Reflecting & evaluating | | - | Data systems | Hold community meetings to understand what the community needs and share progress and results |

^†^ *CFIR: Consolidated Framework for Implementation Research*

^‡^ *ERIC: Expert Recommendations for Implementing Change*

^§^ *CBO: community-based organization*

^¶^ *The ► symbol represents those strategies that were highly feasible and highly impactful*

**Appendix Table 2. Frequency of unique combinations of constructs from the Consolidated Framework for Implementation Research (CFIR) 2.0 by domain and of thematic clusters from the Expert Recommendations for Implementing Change (ERIC)**

| CFIR domain/ ERIC cluster  (N^†^) | Combinations of constructs or clusters | n^‡^ | CBO(s)^§^ |
| --- | --- | --- | --- |
| Outer setting | Partnerships and connections + local attitudes | 2 | 1 |
| Inner setting^¶^  (n = 20) | structural characteristics (work infrastructure) + available resources | 6 | 2 |
|  | structural characteristics + access to knowledge & information | 4 | 2 |
|  | structural characteristics + communications | 4 | 3 |
|  | communications + access to knowledge & information | 2 | 1 |
|  | communications + mission alignment | 2 | 1 |
|  | relational connections + communications | 1 | 1 |
|  | structural characteristics + mission alignment | 1 | 1 |
| Implementation process  (n = 19) | assessing context + engaging + reflecting & evaluating | 2 | 1 |
|  | assessing needs + engaging | 2 | 2 |
|  | assessing needs + engaging + reflecting & evaluating | 2 | 2 |
|  | teaming + assessing context | 2 | 2 |
|  | teaming + reflecting & evaluating + adapting | 2 | 1 |
|  | assessing context + reflecting & evaluating | 1 | 1 |
|  | assessing needs + assessing context | 1 | 1 |
|  | assessing needs + assessing context + planning | 1 | 1 |
|  | assessing needs + reflecting & evaluating | 1 | 1 |
|  | engaging + reflecting & evaluating | 1 | 1 |
|  | planning + tailoring strategies | 1 | 1 |
|  | reflecting & evaluating + adapting | 1 | 1 |
|  | teaming + engaging | 1 | 1 |
|  | teaming + planning | 1 | 1 |
| ERIC thematic clusters  (n = 33) | adapt and tailor to context + change infrastructure | 7 | 3 |
|  | engage consumers + use evaluative and iterative strategies | 6 | 3 |
|  | engage consumers + train and educate relevant groups | 4 | 3 |
|  | develop interrelationships with relevant groups + engage consumers | 2 | 2 |
|  | develop interrelationships with relevant groups + use evaluative and iterative strategies | 2 | 1 |
|  | support staff + train and educate relevant groups | 2 | 1 |
|  | adapt and tailor to context + use evaluative and iterative strategies | 1 | 1 |
|  | change infrastructure + develop interrelationships with relevant groups | 1 | 1 |
|  | change infrastructure + engage consumers | 1 | 1 |
|  | change infrastructure + support staff | 1 | 1 |
|  | change infrastructure + use evaluative and iterative strategies | 1 | 1 |
|  | develop interrelationships with relevant groups + provide interactive assistance | 1 | 1 |
|  | develop interrelationships with relevant groups + train and educate relevant groups | 1 | 1 |
|  | develop interrelationships with relevant groups + support staff | 1 | 1 |
|  | engage consumers + support staff | 1 | 1 |
|  | train and educate relevant groups + use evaluative and iterative strategies | 1 | 1 |
|  |  |  |  |

^†^ *N = total number of strategies mapping to multiple constructs within the domain or multiple ERIC clusters*

^‡^ *n = number of times that unique combination was observed*

^§^ *CBO: community-based organization*

^¶^ *Unless otherwise specified, structural characteristics captures work and/or information technology infrastructure*

**Appendix Table 3. Frequency of unique combinations of Consolidated Framework for Implementation Research (CFIR) 2.0 inner setting and implementation process constructs across strategies and number of community-based organizations (CBOs) for which these combinations were observed**

| Inner Setting Construct | Implementation Process Construct | N | CBOs |
| --- | --- | --- | --- |
| access to knowledge & information | planning | 9 | 3 |
| communications | engaging | 8 | 3 |
| structural characteristics^†^ | reflecting & evaluating | 8 | 3 |
| structural characteristics^†^ | teaming | 8 | 3 |
| structural characteristics^‡^ | planning | 6 | 3 |
| communications | teaming | 5 | 3 |
| structural characteristics^§^ | assessing context | 4 | 2 |
| communications | assessing needs | 3 | 3 |
| structural characteristics^§^ | assessing needs | 3 | 2 |
| structural characteristics^§^ | engaging | 3 | 1 |
| communications | assessing context | 2 | 2 |
| incentive systems | engaging | 2 | 1 |
| relational connections | teaming | 2 | 2 |
| structural characteristics^§^ | tailoring strategies | 2 | 1 |
| access to knowledge & information | engaging | 1 | 1 |
| access to knowledge & information | reflecting & evaluating | 1 | 1 |
| access to knowledge & information | teaming | 1 | 1 |
| available resources | engaging | 1 | 1 |
| available resources | reflecting & evaluating | 1 | 1 |
| available resources | tailoring strategies | 1 | 1 |
| communications | planning | 1 | 1 |
| communications | reflecting & evaluating | 1 | 1 |
| communications | tailoring strategies | 1 | 1 |
| culture | planning | 1 | 1 |
| mission alignment | engaging | 1 | 1 |
| mission alignment | tailoring strategies | 1 | 1 |
| relational connections | assessing context | 1 | 1 |
| structural characteristics^†^ | adapting | 1 | 1 |

**^†^** *Majority of these strategies focused on information technology infrastructure*

**^‡^** *Majority of these strategies focused on work infrastructure*

**^§^** *These strategies aligned with a mix of structural characteristics – both information technology infrastructure and work infrastructure*

**Appendix Figure 1. Comparison of patterns in perceived feasibility and impact of strategies across community-based organizations (CBOs) organized by Consolidated Framework for Implementation Research (CFIR) 2.0 construct and Expert Recommendations for Implementing Change (ERIC) thematic cluster**

|  |  | **Perceived Feasibility^†^** | | | **Perceived Impact^†^** | | |
| --- | --- | --- | --- | --- | --- | --- | --- |
|  |  | **CBO** | | | | | |
|  | **Concept^‡^** | **C** | **B** | **A** | **C** | **B** | **A** |
| **Domain Overview** | Inner only |  |  |  |  |  |  |
|  | Inner + Process |  |  |  |  |  |  |
|  | Outer + Process |  |  | **NA** |  |  | **NA** |
|  | Process only |  | § |  |  | § |  |
| **Inner Setting Domain** | access to knowledge & information |  |  |  |  |  |  |
|  | available resources |  | § |  |  | § |  |
|  | communications |  |  |  |  |  |  |
|  | structural characteristics |  |  |  |  |  |  |
| **Outer Setting Domain** | partnerships & connections |  |  | § |  |  | § |
| **Implementation Process Domain** | assessing needs |  | § |  |  | § |  |
|  | engaging |  |  |  |  |  |  |
|  | planning |  |  |  |  |  |  |
|  | reflecting & evaluating |  | § |  |  | § |  |
|  | teaming |  |  |  |  |  |  |
| **ERIC Thematic Clusters** | Adapt/tailor to context |  |  |  |  |  |  |
|  | change infrastructure |  |  |  |  |  |  |
|  | develop interrelationships with relevant groups and collaborators |  |  |  |  |  |  |
|  | engage consumers |  |  |  |  |  |  |
|  | support staff |  | § |  |  | § |  |
|  | Train/educate relevant groups and collaborators |  |  |  |  |  |  |
|  | use evaluative and iterative strategies |  |  |  |  |  |  |

**^†^ *Dark blue*** *indicates that strategies aligned with that concept were typically considered highly feasible or highly impactful for that specific CBO (≥ 75% of strategies);* ***Light blue*** *indicates that ≥ 65% of strategies aligned with that concept were considered highly feasible or highly impactful;* ***Red*** *indicates that strategies were typically considered less feasible or less impactful for that specific CBO (≥ 75% of strategies);* ***Light red/pink*** *indicates that ≥65% of strategies were considered less feasible or less impactful; White indicates that the perceived feasibility and impact of strategies aligned with the concept is mixed (>35% to <65%)*

**^‡^** *In this table,* ***we only included constructs and clusters aligned with 3 or more strategies for at least two CBOs****. The following concepts were removed because they were only observed for one CBO: inner + outer + process (domain overview), local conditions, critical incidents, external pressure, and financing (outer setting), culture and incentive systems (inner setting), adapting (implementation process), provide interactive assistance (ERIC thematic cluster). The following concepts were removed because at least two CBOs had fewer than three strategies related to the concept: outer only and inner + outer (domain overview), local attitudes (outer setting), mission alignment and relational connections (inner setting), assessing context and tailoring strategies (implementation process), utilize financial strategies (ERIC thematic clusters).*

^§^ *Indicates that the* ***CBO had ≤ 2 strategies*** *related to the concept and therefore patterns should be interpreted with additional caution*

**Appendix Figure 2. Comparison of the distribution of strategies considered highly feasible and highly impactful (Go-Zone) by thematic cluster from the Expert Recommendations for Implementing Change (ERIC) and construct from the Consolidated Framework for Implementation Research (CFIR) 2.0 across community-based organizations (CBOs)^†^**

A. INNER SETTING

|  | access to knowledge & information | available resources | communications | relational connections | structural characteristics |
| --- | --- | --- | --- | --- | --- |
| C | 4 | 1 | 0 | 0 | 4 |
| B | 2 | 1 | 4 | 0 | 3 |
| A | 5 | 0 | 3 | 1 | 4 |

B. IMPLEMENTATION PROCESS

|  | assessing context | assessing needs | engaging | planning | reflecting & evaluating | tailoring strategies | teaming |
| --- | --- | --- | --- | --- | --- | --- | --- |
| C | 1 | 0 | 2 | 2 | 3 | 1 | 1 |
| B | 0 | 1 | 4 | 1 | 0 | 1 | 0 |
| A | 1 | 2 | 2 | 3 | 2 | 1 | 1 |

C. ERIC THEMATIC CLUSTERS

|  | adapt/tailor to context | change infrastructure | develop interrelationships | engage consumers | support staff | train/educate relevant groups | use evaluative and iterative strategies |
| --- | --- | --- | --- | --- | --- | --- | --- |
| C | 1 | 1 | 2 | 2 | 2 | 4 | 3 |
| B | 1 | 1 | 1 | 3 | 0 | 5 | 1 |
| A | 1 | 2 | 2 | 3 | 2 | 6 | 1 |

^†^ *There were too few strategies related to different outer setting determinants to assess feasibility and impact patterns by CBO*

**Appendix Figure 3. Distribution of strategies aligned with domains and constructs from the Consolidated Framework for Implementation Research (CFIR) 2.0 and with thematic clusters from the Expert Recommendations for Implementing Change (ERIC) by feasibility (F) and impact (I) quadrants**

A. DOMAIN OVERVIEW

|  | inner | inner + outer | inner + process | outer | outer + inner + process | outer + process | process |
| --- | --- | --- | --- | --- | --- | --- | --- |
| Low F Lower I | 15 | 1 | 11 | 1 | 0 | 3 | 3 |
| High F Lower I | 5 | 2 | 15 | 2 | 1 | 0 | 1 |
| Low F High I | 9 | 1 | 13 | 0 | 0 | 1 | 1 |
| High F High I | 10 | 1 | 14 | 1 | 1 | 2 | 4 |

B. Outer Setting

|  | critical incidents | external pressure | financing | local attitudes | local conditions | partnerships & connections |
| --- | --- | --- | --- | --- | --- | --- |
| Low F Lower I | 0 | 1 | 0 | 1 | 0 | 4 |
| High F Lower I | 0 | 0 | 0 | 1 | 0 | 4 |
| Low F High I | 0 | 0 | 0 | 2 | 0 | 1 |
| High F High I | 1 | 0 | 1 | 0 | 1 | 2 |

C. Inner Setting

|  | access to knowledge & information | available resources | communications | culture | incentive systems | mission alignment | relational connections | structural characteristics |
| --- | --- | --- | --- | --- | --- | --- | --- | --- |
| Low F Lower I | 3 | 6 | 3 | 0 | 0 | 1 | 2 | 19 |
| High F Lower I | 7 | 1 | 6 | 1 | 1 | 1 | 1 | 8 |
| Low F High I | 1 | 5 | 4 | 1 | 1 | 1 | 0 | 14 |
| High F High I | 11 | 2 | 7 | 0 | 0 | 0 | 1 | 11 |

D. Implementation Process

|  | adapting | assessing context | assessing needs | engaging | planning | reflecting & evaluating | tailoring strategies | teaming |
| --- | --- | --- | --- | --- | --- | --- | --- | --- |
| Low F Lower I | 1 | 3 | 3 | 4 | 2 | 7 | 1 | 6 |
| High F Lower I | 1 | 0 | 0 | 3 | 4 | 3 | 0 | 7 |
| Low F High I | 1 | 2 | 2 | 4 | 4 | 3 | 3 | 3 |
| High F High I | 0 | 2 | 3 | 8 | 6 | 5 | 3 | 2 |

E. ERIC Thematic Clusters

|  | adapt and tailor to context | change infrastructure | develop stakeholder interrelationships | engage consumers | provide interactive assistance | support staff | train and educate stakeholders | use evaluative and iterative strategies | utilize financial strategies |
| --- | --- | --- | --- | --- | --- | --- | --- | --- | --- |
| Low F Lower I | 5 | 15 | 5 | 4 | 0 | 3 | 4 | 7 | 2 |
| High F Lower I | 2 | 5 | 8 | 1 | 0 | 2 | 8 | 3 | 1 |
| Low F High I | 5 | 7 | 4 | 3 | 1 | 0 | 4 | 5 | 3 |
| High F High I | 3 | 4 | 5 | 8 | 0 | 4 | 15 | 5 | 0 |

**Appendix Figure 4. Frequency of Expert Recommendations for Implementing Change (ERIC) thematic clusters organized by Consolidated Framework for Implementation Research (CFIR) 2.0 constructs: CFIR-ERIC matching**

|  | adapt and tailor to context | change infrastructure | develop interrelationships with relevant groups and collaborators | engage consumers | provide interactive assistance | support staff | train and educate relevant groups and collaborators | use evaluative and iterative strategies | utilize financial strategies |
| --- | --- | --- | --- | --- | --- | --- | --- | --- | --- |
| IMPLEMENTATION PROCESS teaming | 6 | 7 | 8 | 0 | 1 | 0 | 1 | 2 | 0 |
| IMPLEMENTATION PROCESS tailoring strategies | 6 | 0 | 0 | 0 | 0 | 0 | 0 | 0 | 1 |
| IMPLEMENTATION PROCESS reflecting & evaluating | 2 | 2 | 2 | 6 | 0 | 0 | 1 | 16 | 0 |
| IMPLEMENTATION PROCESS planning | 1 | 0 | 1 | 0 | 0 | 2 | 12 | 2 | 0 |
| IMPLEMENTATION PROCESS engaging | 0 | 0 | 3 | 13 | 0 | 1 | 7 | 5 | 2 |
| IMPLEMENTATION PROCESS assessing needs | 0 | 2 | 3 | 5 | 0 | 0 | 0 | 5 | 0 |
| IMPLEMENTATION PROCESS assessing context | 1 | 0 | 2 | 2 | 0 | 0 | 0 | 5 | 0 |
| IMPLEMENTATION PROCESS adapting | 0 | 0 | 1 | 1 | 0 | 0 | 0 | 3 | 0 |
| INNER SETTING structural characteristics | 10 | 28 | 5 | 6 | 1 | 7 | 4 | 8 | 1 |
| INNER SETTING relational connections | 0 | 0 | 4 | 0 | 0 | 0 | 0 | 0 | 0 |
| INNER SETTING mission alignment | 1 | 1 | 0 | 0 | 0 | 0 | 1 | 0 | 0 |
| INNER SETTING incentive systems | 0 | 0 | 0 | 0 | 0 | 0 | 0 | 0 | 2 |
| INNER SETTING culture | 0 | 0 | 1 | 0 | 0 | 0 | 0 | 1 | 0 |
| INNER SETTING communications | 2 | 4 | 6 | 6 | 0 | 0 | 6 | 1 | 0 |
| INNER SETTING available resources | 1 | 8 | 1 | 1 | 0 | 0 | 1 | 1 | 3 |
| INNER SETTING access to knowledge & information | 0 | 0 | 1 | 0 | 0 | 3 | 21 | 1 | 0 |
| OUTER SETTING partnerships & connections | 0 | 1 | 7 | 0 | 0 | 0 | 5 | 1 | 0 |
| OUTER SETTING local conditions | 0 | 0 | 1 | 0 | 0 | 1 | 0 | 0 | 0 |
| OUTER SETTING local attitudes | 0 | 0 | 1 | 1 | 0 | 0 | 3 | 0 | 0 |
| OUTER SETTING financing | 0 | 0 | 0 | 0 | 0 | 0 | 0 | 1 | 0 |
| OUTER SETTING external pressure | 0 | 0 | 0 | 0 | 0 | 0 | 0 | 1 | 0 |
| OUTER SETTING critical incidents | 0 | 0 | 0 | 0 | 0 | 0 | 1 | 0 | 0 |
